# Influence of temperature on microbially induced calcium carbonate precipitation for soil treatment

**DOI:** 10.1371/journal.pone.0218396

**Published:** 2019-06-18

**Authors:** Jie Peng, Zhiming Liu

**Affiliations:** 1 Key Laboratory of Ministry of Education for Geomechanics and Embankment Engineering, Hohai University, Nanjing, Jiangsu, China; 2 Geotechnical Research Institute, Hohai University, Nanjing, Jiangsu, China; Guangdong Technion Israel Institute of Technology, CHINA

## Abstract

Microbially induced calcium carbonate precipitation (MICP) is a potential method for improvement of soil. A laboratory study was conducted to investigate the influence of temperatures for soil improvement by MICP. The ureolytic activity experiments, MICP experiments in aqueous solution and sand column using *Sporosarcina pasteurii* were conducted at different temperatures(10, 15, 20, 25 and 30°C). The results showed there were microbially induced CaCO_3_ precipitation at all the temperatures from 10 to 30°C. The results of ureolytic activity experiments showed that the bacterial had higher ureolytic activity at high temperatures within the early 20 hours, however, the ureolytic activity at higher temperatures decreased more quickly than at lower temperatures. The results of MICP experiments in aqueous solution and sand column were consistent with tests of ureolytic activity. Within 20 to 50 hours of the start of the test, more CaCO_3_ precipitation was precipitated at higher temperature, subsequently, the precipitation rate of all experiments decreased, and the higher the temperature, the faster the precipitation rate dropped. The final precipitation amount of CaCO_3_ in aqueous solution and sand column tests at 10 °C was 92% and 37% higher than that at 30 °C. The maximum unconfined compressive strength of MICP treated sand column at 10 °C was 135% higher than that at 30 °C. The final treatment effect of MICP at lower temperature was better than that at high temperature within the temperature range studied. The reason for better treatment effect at lower temperatures was due to the longer retention time of ureolytic activity of bacteria at lower temperatures.

## 1 Introduction

Microbially induced CaCO_3_ precipitation (MICP) via urea hydrolysis has been proposed for soil reinforcement [1–4]. MICP refers to the process in which the metabolites of the urease-producing bacteria react with substance in the surrounding environment to synthesize calcium carbonate (CaCO_3_) [5, 6]. The urease-producing bacteria generate urease during the metabolic process and the urease can catalyze hydrolysis of the urea, which increases the pH of the surrounding solution and forms ammonium ions and carbonate ions (${CO}_{3}^{2-}$ ions) [7, 8]. Because of the negatively charged bacterial surface, calcium ions (Ca^2+^) are adsorbed onto the cell wall surface [9]. When ${CO}_{3}^{2-}$ and Ca^2+^ ions are oversaturated in the surrounding solution, Ca^2+^ and ${CO}_{3}^{2-}$ ions will form CaCO_3_ crystals with cells as the crystal nuclei [10, 11]. CaCO_3_ formed during the MICP process not only can fill the pores in the soil but also is an excellent cementing material that cements soil particles together [4]. Accordingly, the processes improves soil strength [12], reduces the porosity, and decreases the permeability of the soil [13]. The bacteria used in MICP process was nonpathogenic and native to the subsurface environment. Only nutrients and calcium sources are required in MICP process, Treatment methods based on this process are more environmentally friendly than traditional methods. MICP advantages include cheaper treatment in the long run [14], reduced environmental impacts [15], and potential implementation for different applications [9, 16, 17].

Many factors affect the biochemical properties of the MICP bacteria, including temperature [18–21], salinity [22, 23], nutrient type and concentration [24], and calcium source and concentration [7, 25, 26]. Temperature is one of the most important factors to *S*. *pasterurii*'s growth and metabolism. Within a given range, an increase in temperature results in increased growth and activity [20, 27, 28]. Increasing the temperature from 15 to 20 °C increased rate of ureolysis [20, 28]. A linear increase of the ureolytic activity of *S*. *pasteurii* was reported [14] with temperature increase between 25 and 60 °C. Studies have reported on the influence of temperature on the calcium carbonate precipitation of MICP bacteria [29–32]. In the range of 2 to 32 °C, carbonate precipitation is observed, and the precipitation rate increases at higher temperature. Several urease-producing bacteria exhibited [19] calcinogenic activities on Euville limestone at different temperatures (10°C, 20°C, 28°C, 37°C). However, the experiments were conducted using liquid media, agar or limestone, which are not representative for soil conditions. Studies have noted that the urease-producing bacteria have a relatively high activity in the temperature range of 30–70 °C [14, 33]; however, the peak value of the soil temperature is not greater than 30 °C in most regions of the world [34–36]. Except in winter in cold regions, the soil temperature ranges from 10 °C to 30 °C in most regions of the world [37]. In particular, the temperature is about 10 to 16 °C in soils that are 3 to 4 m below the ground surface in most cases. What’s more, in most prior studies on MICP, the bacteria suspension was injected into the soil once and was used for several days to precipitate CaCO_3_. During the storing process or the MICP process (whole life cycle of bacteria), ureolytic activity of bacteria will change significantly. The change of ureolytic activity during whole life cycle of bacteria under different temperatures should not be ignored.

Considering these previous studies and practical soil temperature conditions, the main objective of this study is to investigate the influence of temperature on the change of ureolytic activity during whole life cycle of bacteria and the performance of MICP in soil. The change of ureolytic activity during the storing process were monitored under different temperature (10, 15, 20, 25 and 30°C). To study the feasibility and reinforcing effect of MICP under practical soil temperature conditions, this study investigates MICP in aqueous solution and sand column experiments at 10, 15, 20, 25 and 30 °C. In the experiments in aqueous solution, bacteria-induced CaCO_3_ precipitation rate, and total amount of CaCO_3_ precipitates were measured. In addition, X-ray diffraction (XRD) analysis was conducted on the CaCO_3_ crystals. In the sand column experiments, the strength and amount of precipitated CaCO_3_ of the cemented sand samples were tested and analyzed.

## 2 Material and methods

### 2.1 Bacteria and growth medium

*S*. *pasteurii* (ATCC 11859) was grown at 30°C in a culture medium, which contained the following per liter of deionized water: 10 g NH_4_Cl, 20 g yeast extract, 10 mg MnSO_4_•H_2_O and 24 mg NiCl_2_• 6H_2_O and using 1M NaOH to adjust pH to about 9.0. The ingredients were autoclaved separately and mixed together after sterilization. The culture medium was inoculated with the *S*. *pasteurii* stock culture and incubated aerobically at 30°C in a shaking water bath with 200 rev min^-1^ for approximately 40 h before harvesting at a final optical density (OD_600_,600 nm) of 0.590(about 4.185×10^7^ CFU/mL) [38], and the bacteria were re-suspended in saline solution of 8.5 g/L NaCl. After that, the suspended bacteria was stored at 4 °C until used[39].

### 2.2 Cementation media

A mixed urea-CaCl_2_ solution was used as the cementation media. Urea was the nitrogen source for bacteria growth, and CaCl_2_ was the calcium source during the MICP process. Because reducing the urea to calcium ratio to unity can lower byproduct production and improve calcium carbonate precipitation rate [3], the concentration of the Urea and the CaCl_2_ in this study was set as 1.0M and 0.5M, respectively.

### 2.3 Sand

Ottawa silica sand (U.S. Silica Company) was used in the experiments. The characteristics of Ottawa sand are shown in Table 1.

**Table 1:** Characteristics of Ottawa sand.

Soil type	*D*_50_(mm)	*C*_*u*_	*C*_*c*_	*G*_*s*_	*e*_*min*_	*e*_*max*_	Mineralogy	Shape
Ottawa	0.33	2.2	1.1	2.66	0.47	0.75	Quartz	Round

### 2.4 Tests of ureolytic activity during storing process

To investigate changes of ureolytic activity of the *S*. *pasteurii* untill it died under different temperature, 100 mL stored bacteria was put in 250 mL conical flask with little interference under temperature of 10, 15, 20, 25 and 30°C. Without adding new nutrients, the ureolytic activity would change under different temperature and ending to be 0 while nutrients being consuming and bacteria’s excretion being accumulating (undisturbed metabolism). Conductivity method was used to determine the ureolytic activity of bacteria [14]. One mL bacteria solution was added to 9 mL of 1.11 M urea at corresponding experimental temperature and changes of relative conductivity in the 5-minutes were recorded for 3 times continuously. Average of the 5-minutes relative conductivity was used for ureolytic activity calculation. The bacteria’s ureolytic activity at different temperatures was determined every 12 hours until the it was close to 0.

### 2.5 MICP experiments in aqueous solution

**Fig 1:**
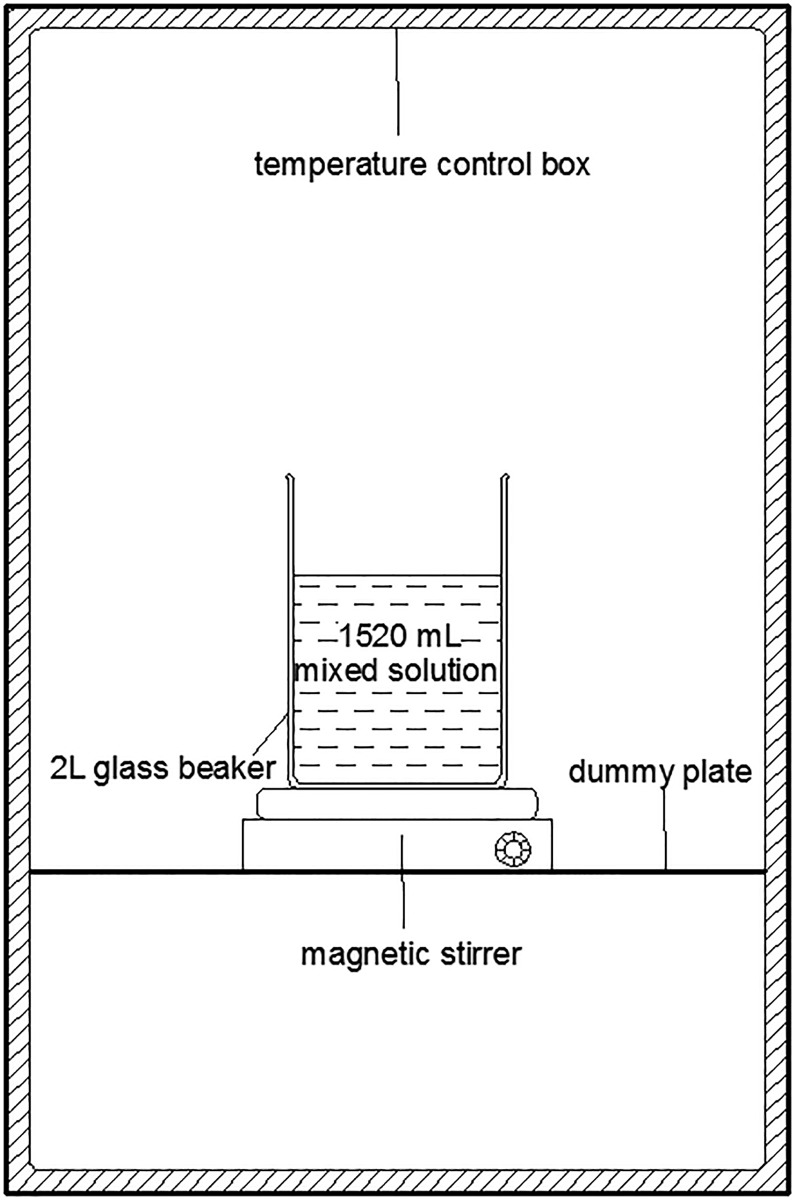
Schematic plan of MICP experiments in aqueous solution.

MICP precipitation experiments in aqueous solution were conducted at 10, 15, 20, 25 and 30 °C. Eighty mL of stored bacteria solution and 1440 mL of cementation media were placed into 2 L aqueous solution at different temperatures. The volume of the cultured bacterial liquid was 5% of the volume of the cementation media, in order to ensure that the cementation media provides sufficient calcium ions during the experiment. The magnetic stirrer was used to ensure the bacteria and cementation media are mixed uniformly. Because some MICP-induced CaCO_3_ were adsorbed on the inside wall of the beaker, it is difficult to measure the total CaCO_3_ precipitation amount directly, so the concentration of the Ca^2+^ ions in the mixed solution was measured to calculate the total CaCO_3_ precipitation amount. The concentration of the Ca^2+^ ions was determined by EDTA titration method [1]. Fig 1 shows the schematic plan of test setup.

### 2.6 Sand column experiments

**Fig 2:**
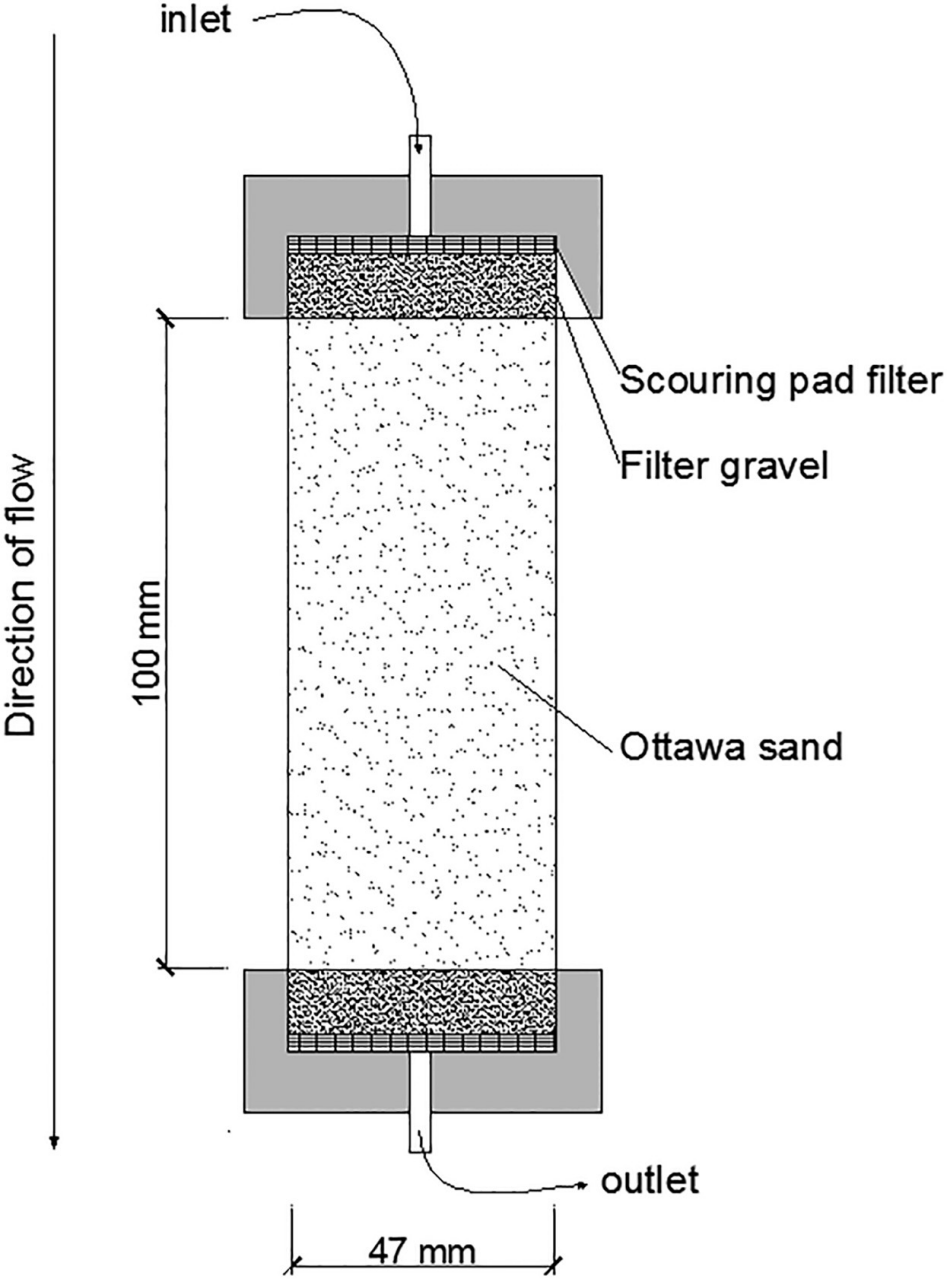
Sand column experiment setup.

To evaluate the feasibility of MICP for improving soil properties under realistic soil temperature conditions, the sand column experiments were conducted at 10, 15, 20, 25 and 30 °C. The columns were made of PVC tubing (length: 100 mm, diameter: 47 mm) (Fig 2). The top and bottom of the column were covered with a layer of 1 cm gravel to serve as a filter and a layer of scouring pad. The middle part of the sand column was 100 mm Ottawa silica sand with characteristics shown in Table 1. The Ottawa sand was packed by tamping to a dry density of approximately 1.63 g/cm^3^ (void ratio of 0.63). The packing was conducted under saturation. The sand column was positioned vertically. A peristaltic pump (Longerpump, BT100-2J, Baoding, China) was connected to the injection point at the top of the sand column to induce flow from top to bottom under a controlled flow rate. Every sand sample was grouted 75mL(about 1 pore volume) 0.05mol/L CaCl_2_, then 75mL bacteria solution 12 hours later, then followed by cementation media(75mL) every 12 hours for 6–20 times.

### 2.7 CaCO_3_ content

In the precipitation experiments in aqueous solution, the calcium carbonate was determined by EDTA method as described in 1.5. In the MICP sand column experiments, the CaCO_3_ content of specimen was determined using the acid washing method. Specimens were crushed using a mortar and oven-dried. The dry soil was washed in HCl solution (0.1 M) to dissolve precipitated CaCO_3_, rinsed, drained, and oven-dried. The difference between the two weights was considered to be the weight of the precipitated CaCO_3_ [14].

### 2.8 Unconfined compression strength (UCS)

The unconfined compression strength is an important index that evaluates the reinforcing effect on a specimen. To study the MICP treatment effect at different temperatures, each sand column specimen was subjected to an UCS test. The specimens of sand column reinforced by MICP for UCS tests were cylinder-shaped with 47-mm diameter and 100-mm height. Before the UCS tests, the sand column samples were sterilized by autoclaving for 20 minutes at a temperature of 121 °C to kill the bacteria. The UCS tests were conducted under strain controlled conditions at a uniform loading rate of 1.5%/min in accordance with ASTM D2166/D2166M-13.

### 2.9 Micro-structural analysis

X-ray powder diffraction analysis(XRD) was done on the CaCO_3_ precipitated at 10 °C,15 °C,20 °C,25 °C and 30 °C in the aqueous solution experiments. With the bacteria concentration of 4.185×10^7^ CFU/mL, the precipitated CaCO_3_ in the aqueous solution was air-dried and grinded to powder. Subsequently, the samples were for XRD analysis to identify the crystalline phase using Ultima IV.

Cheng et al [40] analyzed microstructure of CaCO_3_ crystals in treated sand samples and found that the size of CaCO_3_ crystals varied significantly at temperature of 4 °C,25 °C and 50 °C. However, the influence of temperature on CaCO_3_ crystals formed at practical soil temperature range was not fully investigated. Produced or treated at 10 °C,20 °C and 30 °C, the CaCO_3_ powder and sand samples were air-dried for Scanning Electron Microscopy(SEM) analysis using JSM-7600F SEM.

## 3 Results

### 3.1 Ureolytic activity

**Fig 3:**
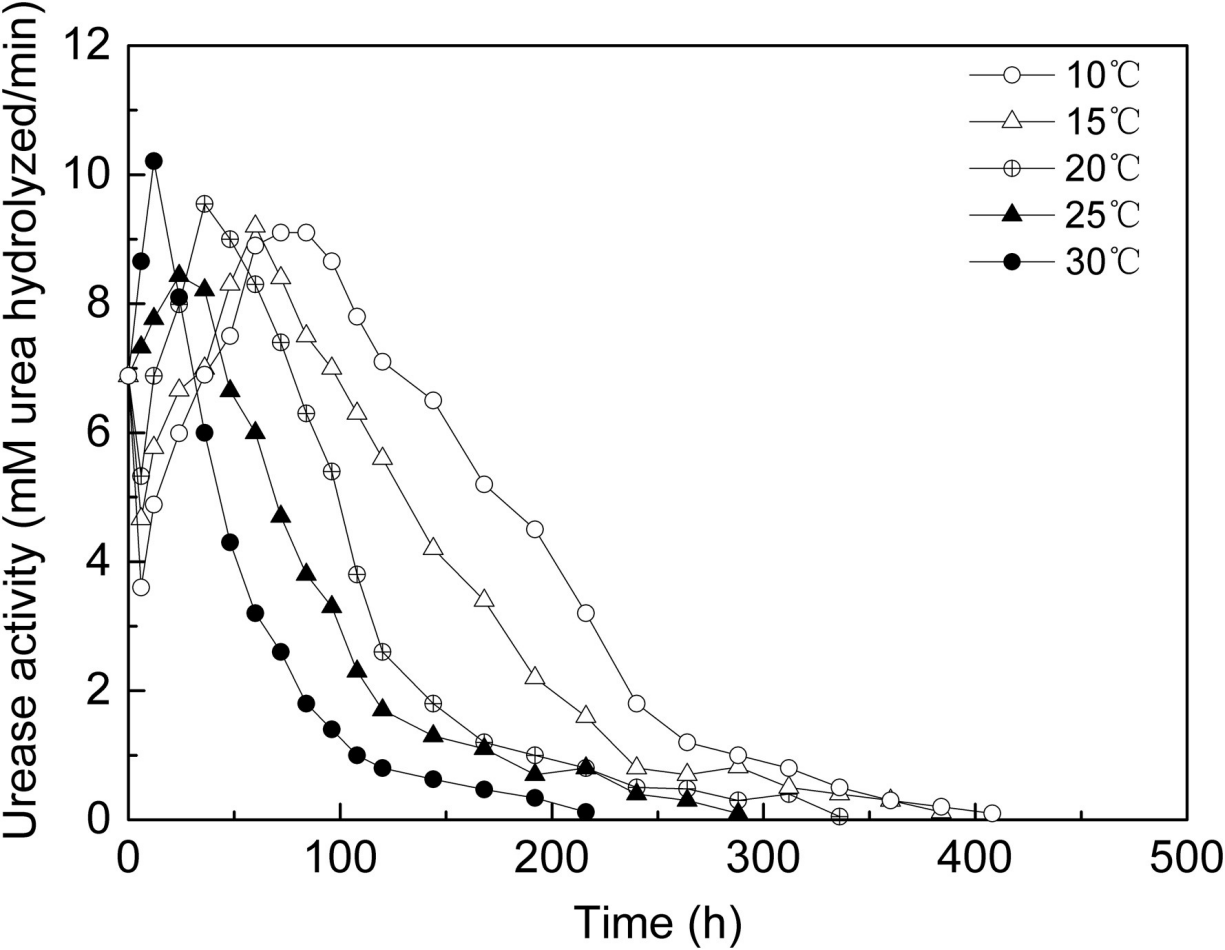
Ureolytic activity vs. time at different temperatures.

Fig 3 shows the ureolytic activity of bacteria vs time at different temperatures. In the first 20 hours, the urease activities at 30 °C and 25 °C increased, the urease activities at 20 °C, 15 °C and 10 °C decreased. The ureolytic activity of bacteria decreased when they were placed from a higher temperature environment to a lower temperature environment. At about 20h after the experiment began, the bacteria ureolytic activity at 30 °C and 25 °C increasing to a peak value and decreased quickly, and from 20h to 50h the urease activities at 20 °C, 15 °C and 10 °C increasing to a peak value, and subsequently, all decreased. The higher the temperature, the faster the ureolytic activity declines. For instance, bacteria kept the ureolytic activity higher than 0.2mS·cm^-1^ for 70h at 30°C,100h at 25°C,130h at20°C,220h at 15°C and 360h at 10°C. After about 220 hours, the ureolytic activity at 30 °C dropped close to 0, that means most bacteria at 30 °C died or lost ureolytic activity. However, the ureolytic activity at 10 °C dropped close to 0 until 400 hours. It is obviously that the lower the experimental temperature, the higher and the longer the ureolytic activity is maintained.

The area enclosed by the ureolytic activity curve and time coordinate, as showed in Fig 3, might reflect the theoretical accumulated hydrolysis capacity of the bacteria at different temperatures, as showed in Table 2. The ratio of the hydrolysis capacity at each temperature is 2.8(10 °C): 2.2(15 °C): 1.7(20 °C): 1.4(25 °C): 1.0(30 °C).

**Table 2:** Total hydrolysis capacity under different temperature.

Temperature	10 °C	15 °C	20 °C	25 °C	30 °C
Area(mS•cm^-1^•h)	39.9	53.9	68.6	88.1	110.4

### 3.2 CaCO_3_ content in precipitation experiments in aqueous solution

**Fig 4:**
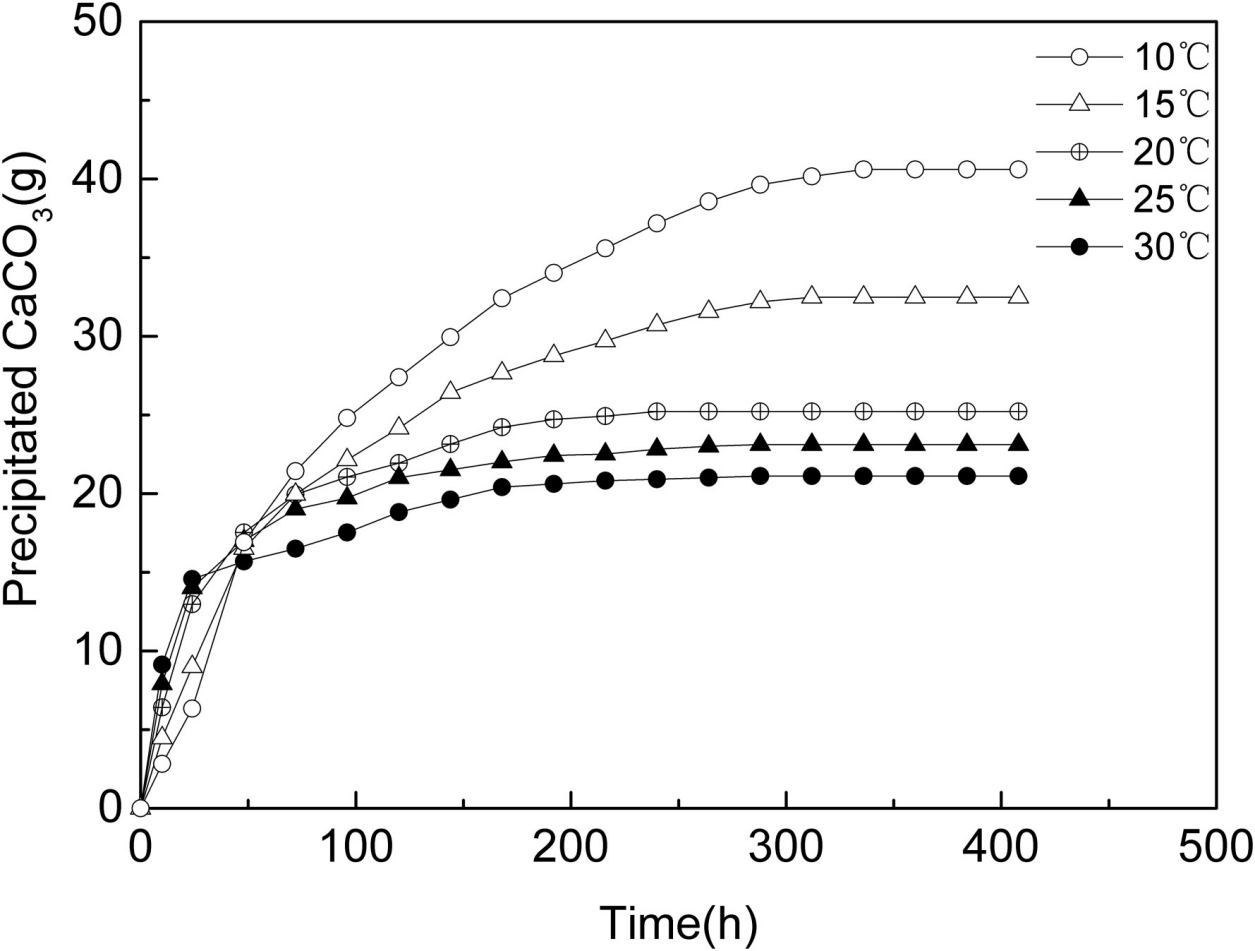
Amount of CaCO_3_ precipitation vs. time.

**Fig 5:**
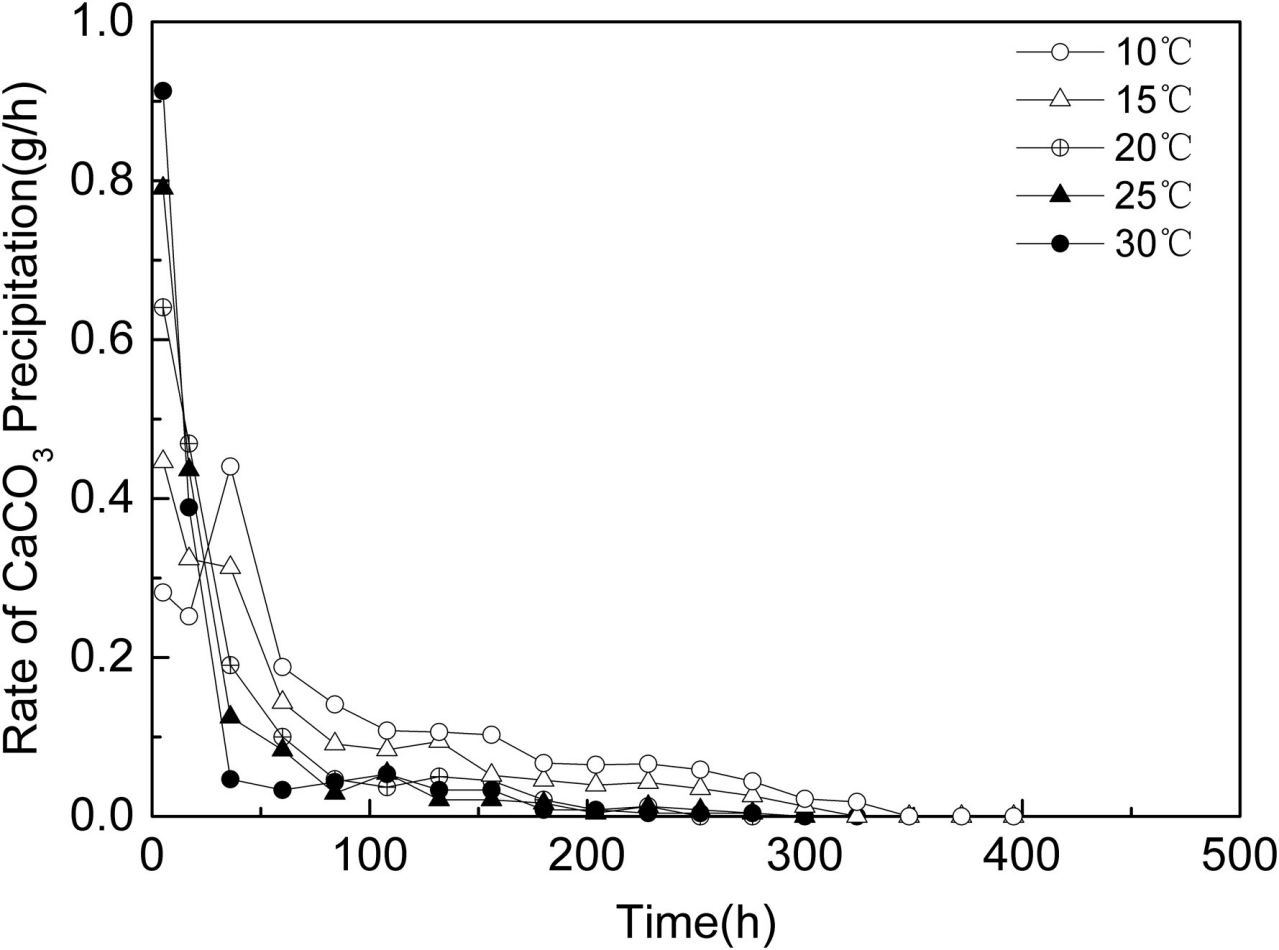
Rate of CaCO_3_ precipitation vs. time.

The amount and rate of precipitation of calcium carbonate at different temperatures are shown in Figs 4 and 5. The results show that during early stage of the experiments (first 30 hours), the higher the temperature, the more the CaCO_3_ precipitated and the higher the precipitation rate was. Subsequently, the precipitation rate of all experiments decreased, the higher the temperature, the faster the precipitation rate dropped. That mean actual reaction time(effective time) of the MICP process was relative longer at lower temperature. What’s more, the precipitation amount of CaCO_3_ at the lower temperatures gradually exceeded the precipitation amount of CaCO_3_ at higher temperatures. When the experiments ended, the precipitated CaCO_3_ amounts at different temperatures were 40.6 g(10 °C), 32.5 g(15 °C), 25.2 g(20 °C), 23.1 g(25 °C) and 21.1 g(30 °C),while the theoretical amount of precipitated CaCO_3_ is about 72g if all CaCl_2_ was consumed.

**Fig 6:**
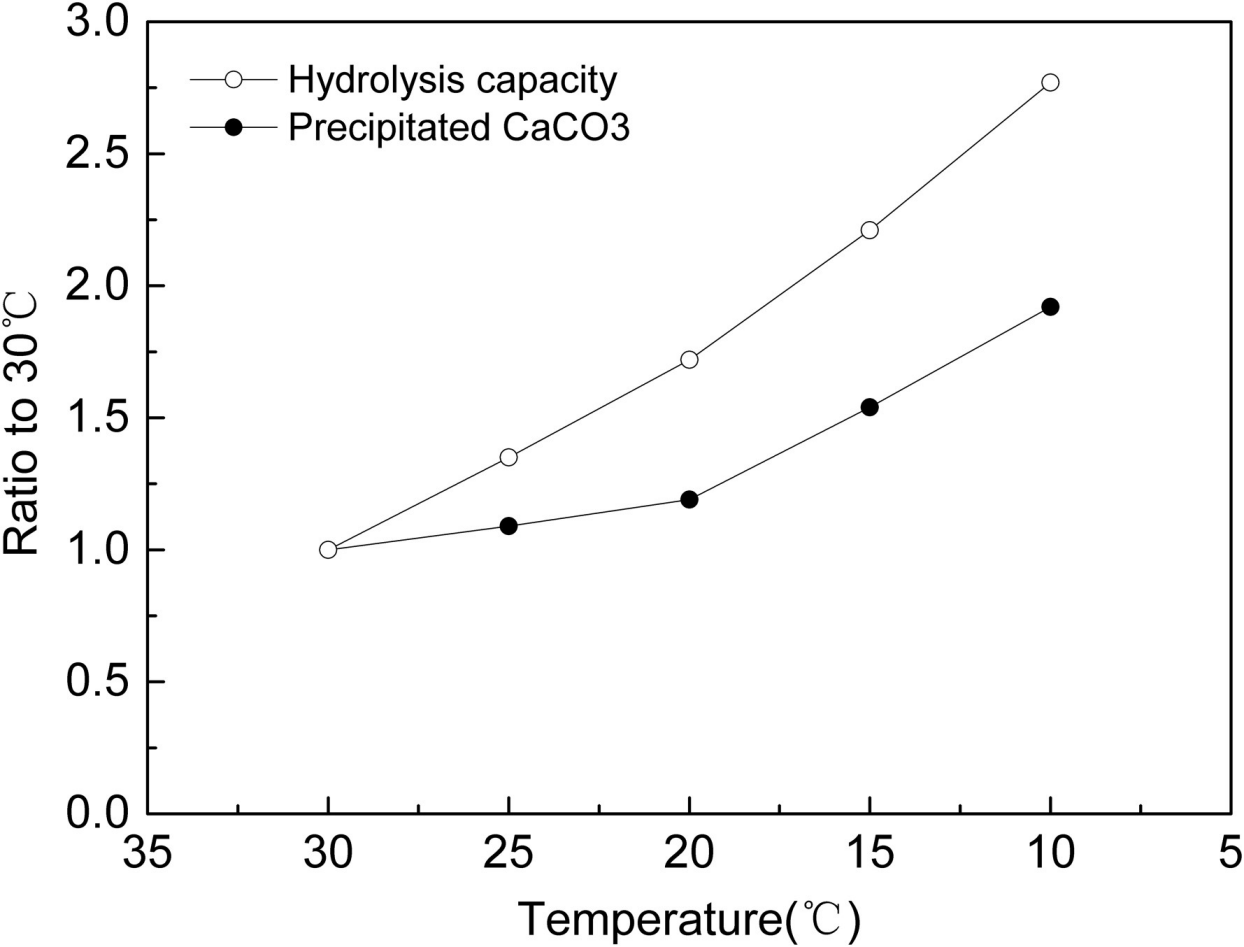
Ratio of hydrolysis capacity and precipitated CaCO_3_ at different temperatures to 30 °C.

Comparing Figs 2 and 3, the trend of bacterial activity at different temperatures is consistent with the trend of the rate of CaCO_3_ precipitation in aqueous solution, the higher the temperature, the higher the initial ureolytic activity but the faster the ureolytic activity declines, therefore, the ability of the bacteria to hydrolyze urea was reduced, and the rate of CaCO_3_ precipitation decreased rapidly. However, the ratio of the hydrolysis capacity in 2.1 was different with the ratio of the amount of precipitated CaCO_3_, as showed in Fig 6. As the temperature decreased, the gap between the hydrolysis capacity of the bacteria and the amount of precipitated CaCO_3_ increased, that was, the lower the temperature, the more ability of bacteria to hydrolyze was not converted to CaCO_3_ precipitation.

### 3.3 Results of Micro-structural analysis

**Fig 7:**
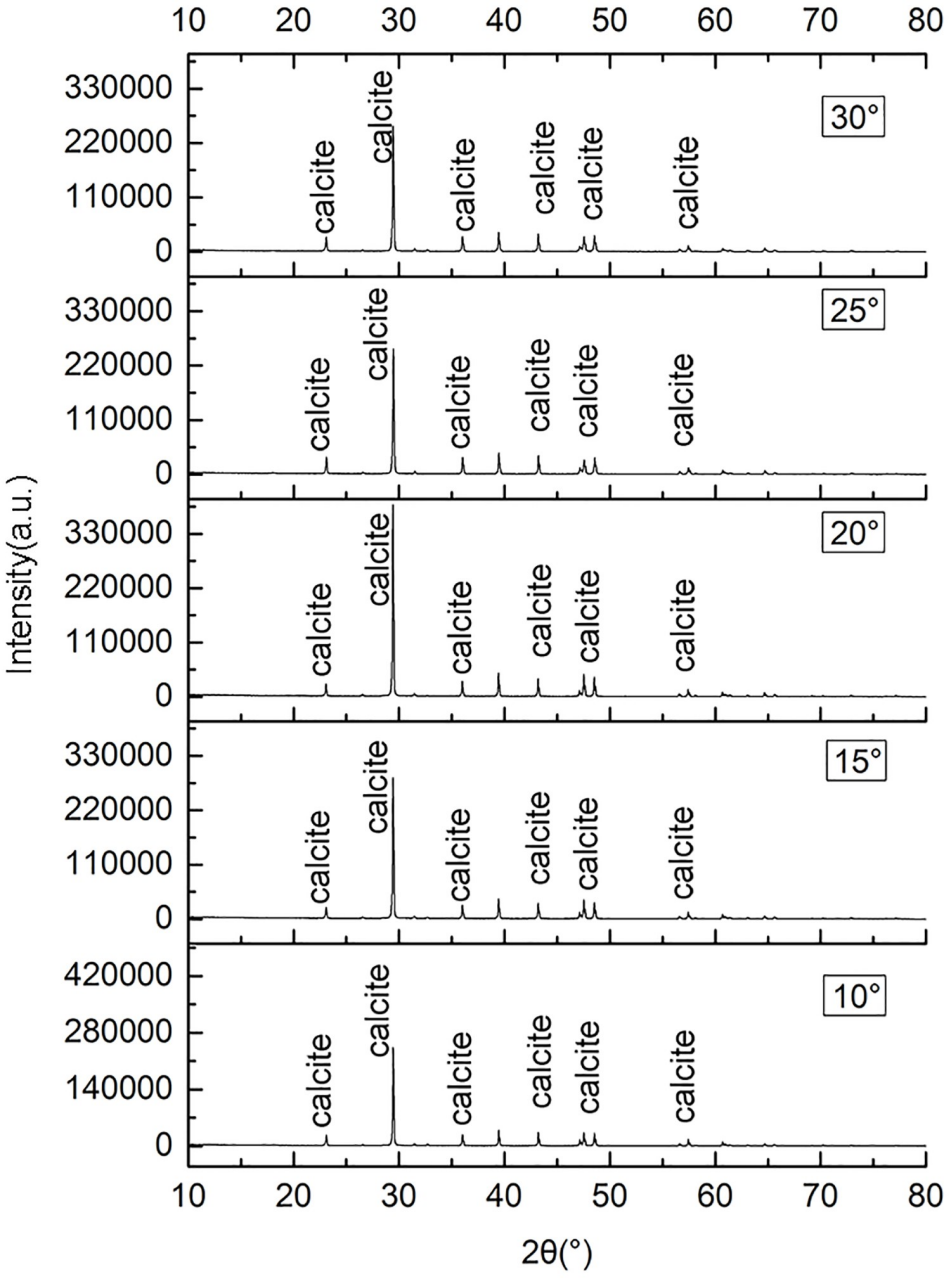
XRD diffraction pattern of the calcium carbonate precipitates produced at different temperatures.

The XRD diffraction pattern of the CaCO_3_ precipitates produced at different temperatures was showed in Fig 7. It showed that the diffraction patterns of the five samples are similar, while the intensity of the peaks among samples varied. The result of XRD analysis showed that the mineral composition is calcite which means the CaCO_3_ produced in the MICP has only one crystalline form of calcite in temperature range of 10 °C -30 °C.

**Fig 8:**
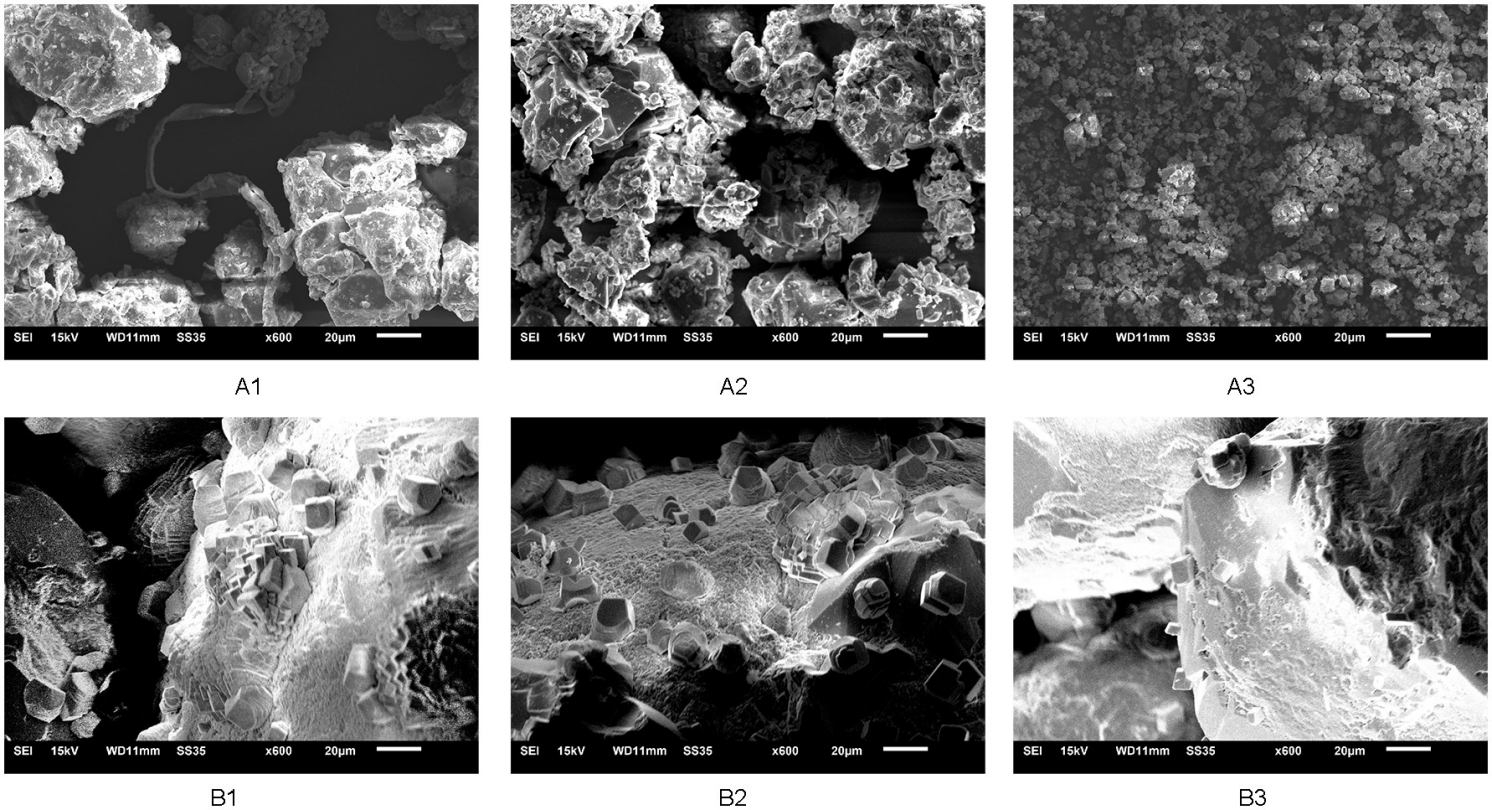
SEM images of CaCO_3_ powder produced at (A1-A3: 10 °C,20 °C and 30 °C) and sand samples treated at (B1-B3: 10 °C,20 °C and 30 °C).

The results of SEM analysis of the samples produced or treated at 10°C, 20 °C and 30 °C were shown in Fig 8. Most crystals of the CaCO_3_ powder was irregularly shaped, while CaCO_3_ crystals adhered to the sand grains were relative regular. The size of the CaCO_3_ crystals decreased gradually as the temperature increased and some large-size crystals were formed at 10°C and 20°C, which was observed in both the CaCO_3_ powder samples and the sand samples. Take the CaCO_3_ powder samples for example, the size of the CaCO_3_ particles formed at 30°C was mainly smaller than 10μm, and the CaCO_3_ particles formed at 10 °C could be huger than 100μm (10 times as that at 30 °C).

### 3.4 UCS of sand column experiments

**Fig 9:**
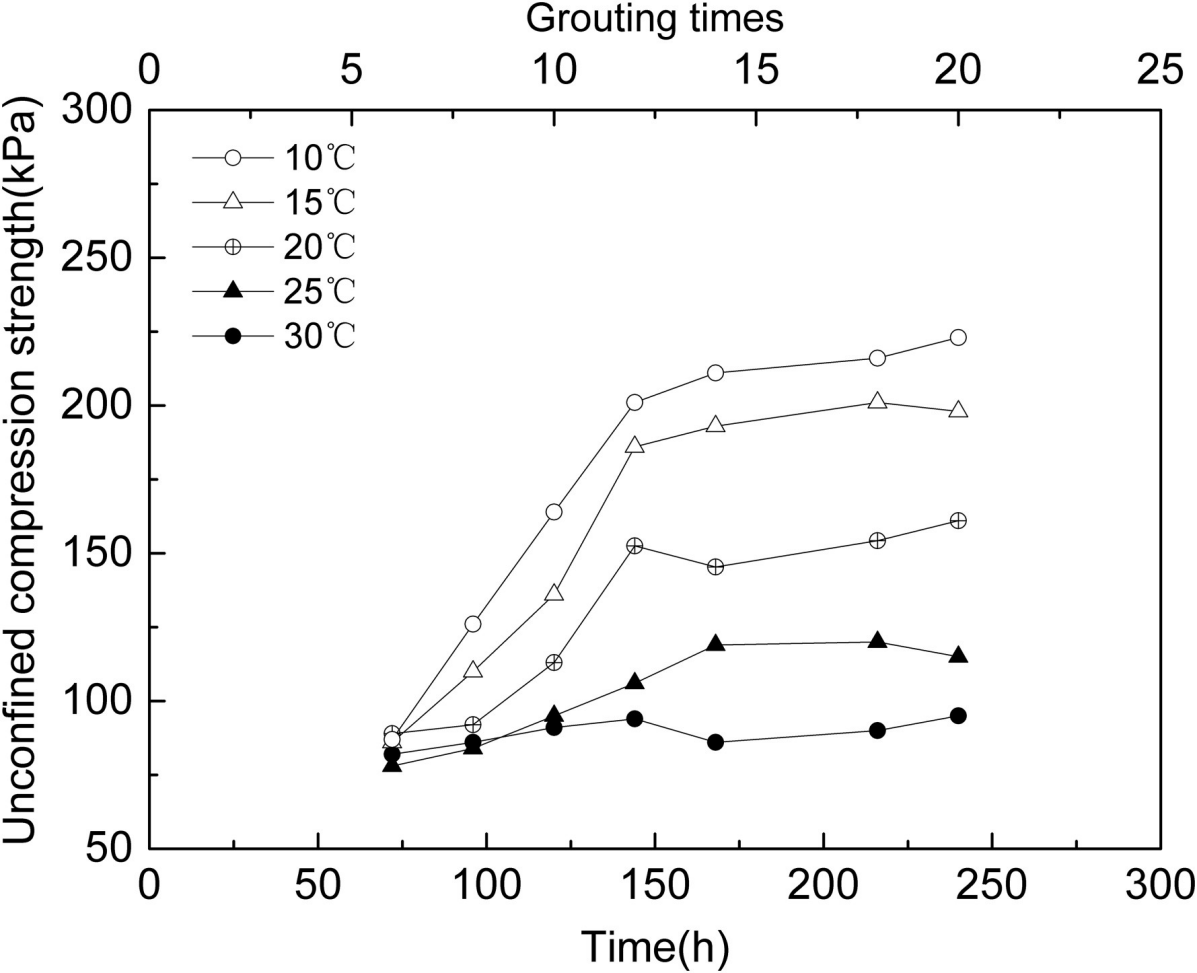
Unconfined compression strength of MICP-treated sand column.

Sand column experiments of MICP were done at different temperatures, the unconfined compression strength over different treatment time(or grouting times) at each temperature was measured as described in 1.8, the results are showed in Table 3 and Fig 9. It can be seen that the UCS of MICP treated sand sample increases with treatment time(or grouting times), and the lower temperature, the higher UCS is. The UCS of the MICP treated sand samples with 6~20 grouting times at 10 °C was 87~223 kPa, but it was 89~161 kPa at 20 °C and it was 82~95 kPa at 30 °C. The UCS of the sand sample decreased with increasing temperatures. The maximum UCS of MICP treated sand column at 10 °C was 223 kPa, 2.35 times that at 30 °C which was 95 kPa.

**Table 3:** Unconfined compression strength at different temperatures over different treatment time(grouting times) (kPa).

Treatment time(h)	Grouting times	30 °C	25 °C	20 °C	15 °C	10 °C
72	6	82	78	89	86	87
96	8	86	84	92	110	126
120	10	91	95	113	136	164
144	12	94	106	152	186	201
168	14	86	119	145	193	211
216	18	90	120	154	201	216
240	20	95	115	161	198	223

### 3.5 CaCO_3_ content in MICP sand column experiments

**Fig 10:**
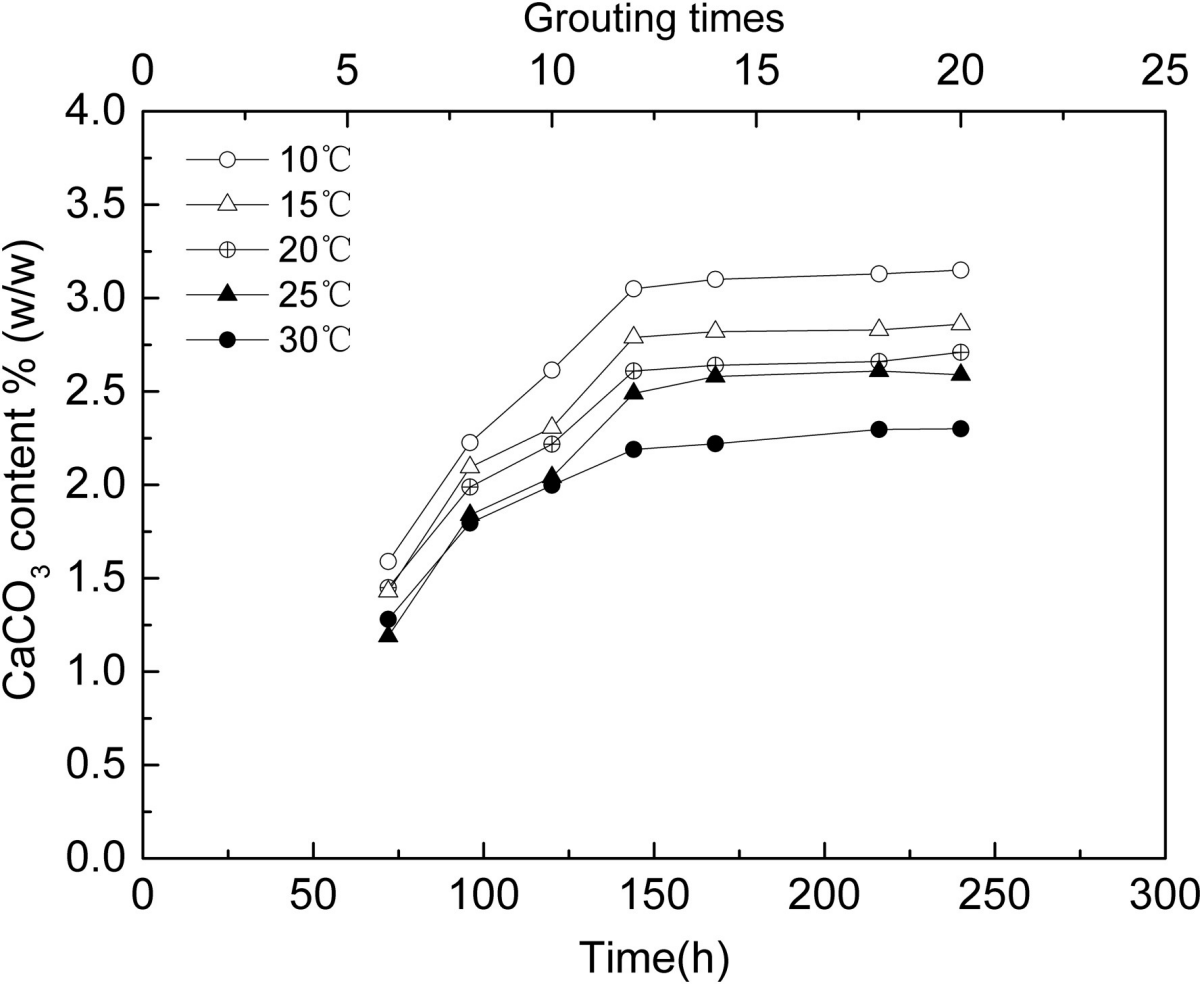
CaCO_3_ content after different treatment times at different temperatures.

The CaCO_3_ content of each MICP treated sand column samples was determined according to the method of 1.7. Fig 10 shows the CaCO_3_ content in sand column samples after 6–20 times grouting at different temperature. From 30 to 10 °C, the lower the temperature, the more the amount of the precipitated calcium carbonate, which is consistent with the variation of UCS at different temperatures.

## 4 Discussion

From previous research, it was known that the ureolytic activity of the bacterial cultures was observed to increase with increasing temperatures, which is consistent with the early stage of the experiments in MICP experiments in aqueous solution in this paper. However, the change of ureolytic activity of the bacterial cultures throughout the whole life cycle was not considered in previous research. In most previous researches on MICP, the bacteria suspension was injected into the soil once and used for several days to precipitate CaCO_3_, and so the change of bacteria’s ureolytic activity in the treatment stage were not able to be measured, but it shouldn’t be ignored. In this paper, the change of bacteria’s ureolytic activity and concentration of Ca^2+^ of MICP tests in aqueous solution under different temperatures could reflect possible change of bacteria’s ureolytic activity in practical application of MICP.

The influence of temperature on MICP was mainly reflected in the following two aspects: biochemical and chemical reaction rate, retention time of bacteria’s ureolytic activity. The most important reactions in MICP were following:

$$CO{\left(NH_{2}\right)}_{2}+2H_{2}O\to 2NH_{4}^{+}+CO_{3}^{2-}$$ (1)

$$Ca^{2+}+CO_{3}^{2-}\to CaCO_{3}\text{(s})$$ (2)

The Eq (1) was the urea hydrolysis reaction catalyzed by the bacteria and was decisive in MICP, the rate of reaction (1) increased with increasing temperatures indicated by previous researchers. However, the bacteria was the key role to Eq (1), the results of ureolytic activity test in this paper showed that the bacteria at high temperatures had more activity in the early stage, but lose the activity faster after several days. Similarly, in the aqueous solution tests, the amount of precipitated CaCO_3_ at lower temperatures was more than that at higher temperatures from 10 to 30 °C.

Similar to the results of this paper, Both species of *Escherichia coli O157*:*H7* and *Salmonella Typhimurium* were believed to survive longer at lower temperature(tropical conditions) than higher temperature(temperate environments) [41]. Cools et al. [42] pointed out that *E*. *coli* and *Enterococcus spp*. could survive remarkably better at 5°C than at 25°C. Habteselassie et al. [43] believed that bacteria cells preference different temperatures for growth and survial, and colder temperature can slow death of cells. It can be concluded that microorganisms survive longer and might can produce more metabolites at lower temperatures than at higher temperatures. Overall, combined those similar reports about the influence of temperature on bacteria and the results of how ureolytic activity of the *S*. *pasteurii* changes over time at different temperatures in this study, it can be concluded that the *S*. *pasteurii* might can maintain relatively higher ureolytic activity in MICP process at lower temperatures. It means that MICP process could form CaCO_3_ crystals more slowly however formed more CaCO_3_ because of longer effective time for the MICP process. It might be the main possible reason for better treatment effect at lower temperature.

As showed in Figs 4 and 5, at lower temperature, the CaCO_3_ was precipitated more slowly but the amount of CaCO_3_ was more than at higher temperature. Meanwhile, as showed in Fig 8, CaCO_3_ crystals formed in aqueous solution and sand sample at lower temperature were huger than those at higher temperature. It had been pointed out that slowly formed biogenic crystals that are tend to have better adherence to sand grains and a higher consolidative effect compared to crystals that are rapidly formed [44]. It means CaCO_3_ crystal slowly formed at lower temperature might be able to bond sand grains together more strongly. What’s more, according to Cheng et al [40], the size of CaCO_3_ crystal formed by MICP at lower temperature(25°C) was much huger than that at higher temperature(50°C),which made the crystals formed at lower temperature more effective for strength improvement. Thus, another possible reason for higher strength of treated sand sample lower temperature might be that the crystals huger and slowly formed at lower temperature contributes more to the UCS than the ones smaller and rapidly formed at higher temperature.

## 5 Conclusion

Microbially induced CaCO_3_ precipitation(MICP) is a complex biochemical process, the objective of this paper was to study the influence of temperatures relevant for practice to MICP. A series of ureolytic activity experiments, MICP experiments in aqueous solution and sand column using the *S*. *pasteurii* were conducted at different temperatures(10, 15, 20, 25 and 30°C).

The result showed that the *S*. *pasteurii* can precipitate CaCO_3_ at the temperatures from 10 to 30°C,which were all calcite. The result of ureolytic activity experiments showed that at the early stage, the ureolytic activity increased with temperature, but then the ureolytic activity at higher temperatures decreased more quickly than at lower temperatures. The results of ureolytic activity experiments showed that the theoretical total hydrolysis capacity of the *S*. *pasteurii* at 10 °C might be 2.77 times that at 30 °C. The results of MICP experiments in aqueous solution and sand column were consistent with ureolytic activity experiments. In the early stage, the higher the temperature, the more the CaCO_3_ was precipitated and the higher the precipitation rate was. Subsequently, the precipitation rate of all experiments decreased, the higher the temperature, the faster the precipitation rate dropped. In the present research, the precipitation amount of CaCO_3_ at 10 °C was 1.92 times and 1.37 times that at 30 °C in MICP aqueous solution and sand column experiments respectively. The maximum UCS of MICP treated sand column at 10 °C was 223 kPa, 2.35 times that at 30 °C which was 95 kPa.

The mainly reason of better MICP treatment effect at lower temperature might be that the bacteria solution has relatively higher ureolytic activity in the long run and can induce more CaCO_3_ in MICP curing process at lower temperatures in the temperature range of 10–30°C. In addition to more CaCO_3_ could be induced at lower temperature, huger size of CaCO_3_ crystal and stronger bonding ability of CaCO_3_ crystals also might be the reason of higher UCS of treated sand samples. Since the soil temperature gradually decreased with depth from the ground surface in the actual engineering site, the non-uniform MICP treatment effect caused by difference of temperature in soil should be further investigated. How to eliminate this kind of non-uniform treatment effect should be considered in further study.
